# Assessing the role of multiple pregnancies in the association between tetralogy of Fallot and assisted reproductive techniques: a path-analysis approach

**DOI:** 10.1186/1750-1172-9-27

**Published:** 2014-02-20

**Authors:** Karim Tararbit, Nathalie Lelong, Lucile Houyel, Damien Bonnet, François Goffinet, Babak Khoshnood

**Affiliations:** 1 Inserm, UMR S953, Epidemiological research on perinatal health, mothers and children health, Port-Royal Maternity, 6ème étage 53 avenue de l’Observatoire, Paris 75014, France; 2 Congenital heart defects surgery unit, Marie Lannelongue Hospital, Le Plessis Robinson 92350, France; 3 Complex congenital heart defects reference center - M3C-Necker, Paris Descartes University, Sorbonne Paris Cité, Paris 75015, France; 4 Port Royal Maternity - Cochin Hospital, Assistance Publique Hôpitaux de Paris, Paris Descartes University, Sorbonne Paris Cité, Paris 75014, France

**Keywords:** Multiple pregnancies, Reproductive techniques, Assisted, Heart defects, Congenital, Tetralogy of Fallot, Epidemiology

## Abstract

**Background:**

Assisted reproductive techniques (ART) are associated with a higher risk of tetralogy of Fallot (TOF) and multiple pregnancies may be associated with a higher risk of congenital anomalies. We assessed the extent to which the association between ART and risk of TOF may be mediated by the higher risk of multiple pregnancies associated with ART.

**Methods:**

We conducted a case–control study using population-based data from the Paris Registry of Congenital Malformations for the period 1987–2009 and a cohort study of congenital heart defects (EPICARD). The study population included 395 cases of TOF and 4104 malformed controls with no known associations with ART. The analysis was based on a path-analysis model using a counterfactual approach, which allows decomposition of the total effect of ART into an indirect effect (that mediated by the association between ART and multiple pregnancies) and a direct effect.

**Results:**

ART (all methods combined) were associated with a 2.6-fold higher odds of TOF after adjustment for maternal and paternal characteristics and year of birth (adjusted OR 2.6, 95% CI, 1.5-4.5). Most (79%) of the effect associated with ART was a direct effect (i.e., not mediated by multiple pregnancies), whereas 21% of the effect of ART was due to its association with multiple pregnancies (i.e., the indirect effect). In vitro fertilization with intracytoplasmic sperm injection was associated with a 3.5-fold higher odds of TOF (adjusted OR 3.5, 95% CI, 1.1-11.2); 11% of this effect was mediated through the association of ICSI with multiple pregnancies.

**Conclusions:**

By far, most of the higher risk of TOF associated with ART is a direct effect and only a small proportion of the effect may be mediated by multiple pregnancies.

## Background

Assisted reproductive techniques (ART) are methods used to achieve pregnancy in case of female and/or male infertility. ART are known to be associated with a higher risk of multiple pregnancies [1,2], mostly due to superovulation or transfer of two or more eggs or embryos. An overall higher risk of congenital malformations in fetuses conceived following ART has been reported in several studies [3-10] using different study populations and designs and methods of analysis and at times inconsistent results, particularly for specific anomalies [6].

Few studies have assessed specifically the risk of congenital heart defects (CHD) associated with ART [6,11-15]. These studies have found an increased risk of 30-50% (Odds ratios ~1.3-1.5) of the overall risk of CHD, which varied across categories of CHD and methods of ART [12-15]. In particular, a recent study assessing the risk for four individual CHD, (Transposition of Great Arteries, Hypoplastic Left Heart, Coarctation of Aorta and Tetralogy of Fallot (TOF)) [15] found a specifically higher risk of TOF associated with ART. In contrast, the authors found no statistically significant associations with the other three defects examined and the effect sizes (odds ratios) were close to the null value (i.e., close to one).

Even if multiple pregnancies may be independently associated with a higher risk of congenital anomalies [16,17] and this may be specifically the case for CHD, relatively little information exist on the specific association between multiple pregnancies and CHD [18-20], particularly for individual defects [21].

Given: i) the previous finding of the specific association between ART and TOF [15], ii) the known association between ART and multiple pregnancies, and iii) the possible association of the latter with the risk of congenital anomalies, the objective of the present study was to assess the impact of multiple pregnancies in the association between TOF and ART using a path-analysis model.

## Material and methods

### Data sources

Two sources of data were used for this study: 1) the Paris Registry of Congenital Malformations and 2) the EPICARD study (Epidemiological study on the outcomes for congenital heart diseases). These two sources of data are briefly described below.

#### The Paris Registry of Congenital Malformations

Since 1981, the Paris Registry of Congenital Malformations registers all cases of birth defects and chromosomal anomalies among live-births, still-births (≥ 22 weeks of gestation) and termination of pregnancy for fetal anomaly. The Registry covers the population of women who live in Greater Paris area (Paris and its surrounding suburb) and deliver or have a termination of pregnancy for fetal anomaly in a Parisian maternity unit. The annual number of deliveries in our population is about 38,000.

The Paris Registry is a member of the European network of registries of congenital malformations (EUROCAT) and of the International clearinghouse for birth defects surveillance and research [22-25]. The Registry follows the EUROCAT methodology and quality of data is routinely monitored by both EUROCAT and the National committee of registries in France. Review of procedures regarding confidentiality of data is overseen by both the National committee of registries and the National committee of informatics and freedom. Data are based on medical records and are collected from several sources including maternity units, neonatology wards, cytogenetic and pathology services.

In the present study, data from the Registry corresponded to the period 1987 to 2009 as the first case of a malformation with exposure to IVF occurred in 1987 and 2009 was the last year for which data were available at the time of the study.

#### EPICARD

The EPICARD study [26] is an ongoing prospective cohort study of all children with a CHD born to women living in the Greater Paris area (Paris and its surrounding suburbs) between 2005 and 2008 regardless of place of delivery (total number of births 317538). The principal objectives of the study are to use population-based data from a large cohort of patients with CHD to: i) estimate total and live birth prevalence, pre- and postnatal diagnosis of CHD; ii) assess medical and surgical management of children with CHD, iii) evaluate neonatal mortality and morbidity and neuro-developmental outcomes of children with CHD; and iv) identify the factors associated with their health outcomes, especially the role of events during the neonatal period and of the initial medical and surgical management. All cases (live births, terminations of pregnancy for fetal anomaly, fetal deaths) diagnosed in the prenatal period or up to one year of age in the birth cohorts between May 1st 2005 and April 30th 2008 were eligible for inclusion. The total number of cases included in the study was 2867, including 2348 newborns (82%), 466 terminations of pregnancy for fetal anomaly (16.2%) and 53 fetal deaths (1.8%). Diagnoses were confirmed in specialized paediatric cardiology departments and for the majority of terminations of pregnancy for fetal anomaly and fetal deaths by fetopathologist examination; for others in which a pathology exam could not be done (26%) the diagnoses were confirmed by consensus by a paediatric cardiologist and a specialist in echocardiography based on results of prenatal echocardiography examination.

### Methods

We conducted a case–control study with malformed controls. Cases were fetuses/newborns with tetralogy of Fallot (TOF). Two sources of data, the EPICARD study and the Paris Registry of Congenital Malformations (1987–2009), were used for inclusion of cases. The data source for the malformed controls was the Paris Registry data (1987–2009, i.e., the same period as for the cases). Malformed controls were isolated cases of single malformations (i.e. those that were not associated with chromosomal or any other anomalies / genetic syndromes) and for which no evidence of an association with ART was found in the literature. As recommended by Hook [27], we selected a wide spectrum of heterogeneous birth defects as controls in order to decrease the risk of selection bias due to shared etiologic factors between cases and controls. The malformations in the control group comprised club-foot, angioma, skin abnormalities, polydactyly, syndactyly and congenital hip dislocation.

Exposure to ART included the following categories: inductors of ovulation only (IO), in vitro fertilization (IVF) only and IVF with intracytoplasmic sperm injection (IVF + ICSI). Exposure to ART was assessed as: i) a binary variable (ART yes/no), ii) a variable in four categories (no ART, IO, IVF only, IVF + ICSI) and iii) a variable combining IVF only and IVF with ICSI (IVF +/- ICSI) in a single category.

Potential confounding factors considered were maternal characteristics (age, occupation and geographic origin), paternal age and year of birth (or termination of pregnancy for fetal anomaly). Although their exact relations to the risk for specific CHD are not well known, these factors are associated with both exposure to ART and prevalence of birth defects in general [28,29]. Maternal occupation was coded in five categories (professional, intermediate, administrative/public service, other and none) following the French National Institute of Statistics and Economic Studies (INSEE) classification. Geographic origin was coded in four categories: French, North African, Sub-Saharan African and other countries.

### Statistical analysis

**Figure 1:**
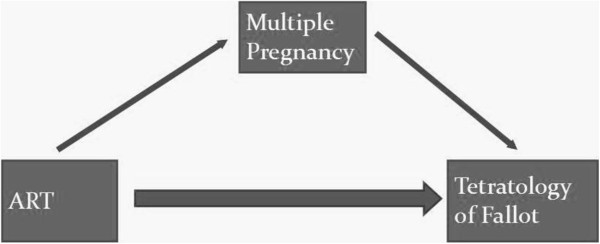
Decomposition of the total effect of ART on the risk of TOF into a direct effect and an indirect effect mediated through multiple pregnancies.

We conducted a logistic regression analysis using a path-analysis model [30,31] to decompose the total effect associated with ART into an indirect effect (that mediated by the association between ART and multiple pregnancies) and a direct effect (Figure 1). The path-analysis model uses a counterfactual (“what if”) approach and its basis may be formulated conceptually as the response to the following question: “What would be the risk of TOF associated with ART if ART-conceived fetuses had the same probability of multiple pregnancies as spontaneously-conceived fetuses?”

In case of a binary exposure, two estimates of the direct and indirect effects are possible. The first one is based on the answer to the counterfactual question above. The second estimate is based on the alternative question: “What would be the risk of TOF associated with ART if spontaneously-conceived fetuses had the same probability of multiple pregnancies as ART-conceived fetuses?” These two estimates are not necessarily the same, although they are usually very similar. One suggestion is to report the average of these two estimates for assessing the direct vs. indirect (mediated) effect of the exposure. In our study the two set of estimates were very similar and we reported the estimates that corresponded to the first counterfactual question noted above.

Analyses were conducted for all cases of TOF and for “isolated” cases of TOF (i.e. excluding cases associated with chromosomal anomalies or anomalies of other systems).

The statistical significance level was set at α = 0.05 and all tests were two-sided. Analyses were done with Stata 11 software (Statacorp, Texas, USA) and add-on models developed by Buis [30].

### Ethics

No specific ethical approval was needed for this particular analysis. The French National Committee of Informatics and Freedom (CNIL) has authorised the surveillance and research activities of the Registry using anonymous data and has approved the EPICARD study.

## Results

### Study population

The study population included 404 cases of TOF, among which 97.8% (n = 395) had complete data on ART. Associated chromosomal anomalies were found in 20.3% (n = 80) of the cases of TOF. The control group included 4250 fetuses, among whom 96.6% (n = 4104) fetuses had complete data on ART. The control group included 35.0% (n = 1 436) with congenital hip dislocation, 20.0% (n = 824) with club-foot, 19.1% (n = 782) with polydactyly, 12.6% (n = 517) with angioma, 9.3% (n = 38) with skin abnormality, and 4.0% (n = 164) with syndactyly.

Table 1 shows the comparison of the characteristics of cases of TOF and controls. Cases and controls were different for most characteristics. In particular, mothers of cases of TOF were older than mothers of controls (31.7 vs. 30.4 years, p < 0.001), and were more likely to be from North Africa (18.1 vs. 10.4%, p < 0.001). Stillbirths and terminations of pregnancy for fetal anomaly were more likely to occur for cases of TOF than controls (4.5 vs. 0.2% and 29.0% vs. 0.3% respectively, p < 0.001). Cases of TOF were more likely to have been conceived following ART (all methods combined) than controls (6.6 vs. 3.5%, p = 0.002). Exposure to the different methods of ART also differed significantly between cases and controls. In particular, 1.3% of cases of TOF were conceived following IVF + ICSI vs. 0.3% of controls (p < 0.001). The study population included 329 cases of TOF and 2670 controls conceived spontaneously. The odds of TOF was 1.5-fold higher (OR= 1.5, 95% CI 0.8-2.7) in twins vs. singleton pregnancies conceived spontaneously; however, this association was not statistically significant (p = 0.17).

**Table 1:** Associations between predictor variables and case/control status

**Characteristics**		**TOF (N=404)**		**Controls (N=4 250)**		**p**
		n*	**%**^**§**^	n*	**%**^**§**^	
**Mother**	*Age* (years)					
	Mean (SD)	31.7 (5.6)		30.4 (5.2)		<0.001
	Median (p25-p75)	31.5 (28 – 36)		30 (27 – 34)		
	<20	3	0.8	59	1.4	<0.001
	20 – 29	146	36.5	1,809	42.8	
	30 – 34	126	31.5	1,434	33.9	
	35 – 39	91	22.8	722	17.1	
	≥ 40	34	8.5	203	4.8	
	*Geographic origin*					
	France	200	50.4	2,412	57.9	<0.001
	North Africa	72	18.1	433	10.4	
	Subsaharan Africa	39	9.8	550	13.2	
	Other	86	21.7	770	18.5	
	*Occupation*					
	None	113	30.9	1,083	26.3	0.096
	Professional	84	23.0	997	24.2	
	Intermediate	64	17.5	856	20.8	
	Administrative/public service	67	18.3	852	20.7	
	Other	38	10.4	330	8.0	
**Father**	*Age* (years)					
	Mean (SD)	35.0 (6.6)		33.9 (6.6)		0.006
	Median (p25-p75)	34 (30 – 39)		33 (29 – 38)		
	<20	1	0.3	5	0.1	0.014
	20 – 29	59	19.5	890	25.8	
	30 – 34	94	31.1	1,198	34.7	
	35 – 39	79	26.2	734	21.3	
	≥ 40	69	22.9	734	21.3	
	*Geographic origin*					
	France	179	49.2	2,325	58.6	<0.001
	North Africa	66	18.1	416	10.5	
	Subsaharan Africa	38	10.4	529	13.3	
	Other	81	22.3	700	17.6	
	*Occupation*					
	None	24	7.6	281	7.4	0.014
	Professional	110	34.6	1,474	38.6	
	Intermediate	44	13.8	566	14.8	
	Administrative/public service	28	8.8	479	12.5	
	Other	112	35.2	1,020	26.7	
**Pregnancy**	*Outcome*					
	Still-births	18	4.5	7	0.2	<0.001
	Live-births	269	66.6	4,231	99.6	
	Pregnancy terminations	117	29.0	12	0.3	
**Art**	None	369	93.4	3,959	96.5	0.004
	IO^$^ only	11	2.8	77	1.9	
	IVF^£^ only	10	2.5	55	1.3	
	IVF+ICSI^¥^	5	1.3	13	0.3	

^*^n represents the number of cases or controls without missing data.

^§^% calculated with the number of cases or controls without missing data as a denominator.

^$^Inductors of ovulation.

^£^In vitro fertilization.

^¥^IVF with intracytoplasmic sperm injection.

Table 2 shows the comparison of the study population characteristics according to exposure to ART (all methods combined). Mothers who had conceived following ART were older than mothers who had conceived spontaneously (33.0 vs. 30.4 years, p < 0.001), more likely to be of French origin (72.9 vs. 56.4%, p < 0.001) and in the highest occupational category “professional” (35.5 vs. 23.6%, p < 0.001). Multiple births were more likely to occur after ART conception (23.7 vs. 2.9% for twins and 4.6 vs. 0.1% for triplets, p < 0.001). Still-births also tended to be more likely in the ART conceived fetuses (1.8 vs. 0.5%, p = 0.099).

**Table 2:** Comparison of the study population characteristics according to exposure to ART (all methods combined)

**Variable**		**Exposure to ART**				**p**
		**No**		**Yes**		
		**n**	**%**^**§**^	**n**	**%**^**§**^	
**Mother**	*Age* (years)					
	Mean (SD)	30.4 (5.2)		33.0 (4.6)		<0.001
	Median (p25–p75)	30 (27 – 34)		33 (30 – 37)		
	<20	61	1.4	0	0.0	<0.001
	20 – 29	1,874	43.4	39	22.8	
	30 – 34	1,440	33.4	68	39.8	
	35 – 39	727	16.9	52	30.4	
	≥ 40	213	4.9	12	7.0	
	*Geographic origin*					
	France	2,416	56.4	124	72.9	<0.001
	North Africa	484	11.3	11	6.5	
	Subsaharan Africa	573	13.4	9	5.3	
	Other	808	18.9	26	15.3	
	*Occupation*					
	None	1,134	27.1	24	14.2	<0.001
	Professional	988	23.6	60	35.5	
	Intermediate	851	20.3	43	25.4	
	Administrative/public service	867	20.7	28	16.6	
	Other	346	8.3	14	8.3	
**Father**	*Age* (years)					
	Mean (SD)	33.9 (6.6)		35.0 (5.8)		0.044
	Median (p25–p75)	33 (29 – 38)		34 (31 – 38)		
	<20	6	0.2	0	0.0	<0.001
	20 – 29	924	26.1	17	10.8	
	30 – 34	1,206	34.0	66	42.0	
	35 – 39	756	21.3	47	29.9	
	≥ 40	652	27.0	27	17.2	
	*Geographic origin*					
	France	2,327	57.1	118	71.5	0.002
	North Africa	463	11.4	11	6.7	
	Subsaharan Africa	550	13.5	11	6.7	
	Other	736	18.1	25	15.2	
	*Occupation*					
	None	291	7.5	6	3.7	0.023
	Professional	1,454	37.7	80	49.1	
	Intermediate	573	14.9	25	15.3	
	Administrative/public service	480	12.4	14	8.6	
	Other	1,060	27.5	38	23.3	
**Pregnancy**	*Outcome*					
	Still-births	22	0.5	3	1.8	0.099
	Live-births	4,183	96.3	163	95.3	
	Pregnancy terminations	123	2.8	5	2.9	
	Multiplicity					
	Singletons	2,908	97.0	94	71.8	<0.001
	Twins	88	2.9	31	23.7	
	Triplets	3	0.1	6	4.6	

^§^% calculated with the total of cases or controls without missing data as a denominator.

### Risk of TOF associated with ART: direct and indirect (mediated by multiple pregnancies) effects

#### All cases of TOF

Table 3 summarizes the results of logistic regression analyses of the association between TOF and ART, including the path-analysis estimates for the decomposition of the total effect of ART into its direct and indirect (mediated by multiple pregnancies) components.

**Table 3:** Decomposition of the total effect of ART on the odds of TOF into a direct effect and indirect effect mediated through multiple pregnancies

**Art**	**Total effect**				**Direct effect**				**Indirect effect**				**Estimated size of the indirect effect**
	Unadjusted OR*	**95% CI**	Adjusted§ OR*	**95% CI**	Unadjusted OR*	**95% CI**	Adjusted OR*	**95% CI**	Unadjusted OR*	**95% CI**	Adjusted OR*	**95% CI**	
None	1.0	ref.	1.0	ref.	1.0	ref.	1.0	ref.	1.0	ref.	1.0	ref.	
All methods combined	1.9	1.2 – 3.1	2.6	1.5 – 4.5	1.7	1.0 – 2.8	2.1	1.2 – 3.7	1.1	1.0 – 1.4	1.2	1.0 – 1.5	20.9%
IO^$^ only	1.4	0.7 – 2.9	2.1	1.0 – 4.7	1.3	0.7 – 2.6	1.8	0.9 – 3.9	1.1	1.0 – 1.2	1.2	1.0 – 1.3	19.1%
IVF^£^ only	2.2	1.0 – 4.7	2.8	1.2 – 6.7	1.8	0.8 – 3.8	2.1	0.9 – 5.2	1.2	0.9 – 1.6	1.3	1.1 – 1.7	27.8%
IVF+ICSI^¥^	3.1	1.0 – 10.0	3.5	1.1 – 11.2	2.8	0.9 – 8.9	3.1	0.9 – 10.1	1.1	1.0 – 1.3	1.1	0.9 – 1.4	11.1%
IVF +/- ICSI	2.4	1.3 – 4.6	3.0	1.6 – 5.8	2.1	1.0 – 4.3	2.4	1.2 – 4.6	1.2	0.9 – 1.5	1.3	1.0 – 1.6	22.3%

*Odds ratios (OR) represent the odds of a birth (including live births, stillbirths and pregnancy terminations) with TOF (cases) relative to the odds of a birth with one of the malformed controls (club-foot, angioma, skin abnormality, polydactyly, syndactyly, and congenital hip dislocation).

^§^Adjustment was made for maternal age, occupation, geographic origin, paternal age and year of birth.

^$^Inductors of ovulation.

^£^In vitro fertilization.

^¥^IVF with intracytoplasmic sperm injection.

ART (all methods combined) were associated with a significant increase in the odds of TOF (total effect: adjusted OR 2.6, 95% CI 1.5 – 4.5). The ART conceived fetuses would have had a 2.1-higher odds of TOF than spontaneously conceived fetuses if the risk of multiple pregnancies were kept constant at the level of spontaneously conceived fetuses (direct effect: adjusted OR 2.1, 95% CI 1.2 – 3.7). This estimates suggested in turn that about 21% of the overall higher odds of TOF associated with ART was due to the higher probability of multiple pregnancies following ART.

IO were associated with a 2.1-higher odds of TOF (total effect: adjusted OR 2.1, 95% CI 1.0 – 4.7). The IO conceived fetuses would have had a 1.8-higher odds of TOF than spontaneously conceived fetuses if they had the same risk of multiple pregnancies as for spontaneously conceived fetuses (direct effect: adjusted OR 1.8, 95% CI 0.9 – 3.9). Hence, about 19% of the overall higher odds of TOF associated with IO was due to the higher probability of multiple pregnancies following IO.

IVF only was associated with a 2.8-higher odds of TOF (total effect: adjusted OR 2.8, 95% CI 1.2 – 6.7). Approximately 28% of this overall higher odds of TOF associated with IVF only was due to the higher probability of multiple pregnancies following IVF only.

Finally, IVF + ICSI was associated with a 3.5-higher odds of TOF (total effect: adjusted OR 3.5, 95% CI 1.1 – 11.2). The IVF + ICSI conceived fetuses would have had a 3.1-higher odds of TOF than spontaneously conceived fetuses if they had the same risk of multiple pregnancies as for spontaneously conceived fetuses (direct effect: adjusted OR 3.1, 95% CI 0.9 – 10.1). Therefore, about 11% of the overall higher odds of TOF associated with IVF + ICSI was due to the higher probability of multiple pregnancies following IVF + ICSI.

#### Isolated cases of TOF

Table 4 summarizes the results of logistic regression analyses of the association between isolated cases of TOF (excluding cases associated with chromosomal or other anomalies), including the path-analysis estimates for the decomposition of the total effect of ART into its direct and indirect (mediated by multiple pregnancies) components..

**Table 4:** Decomposition of the total effect of ART on the odds of TOF without associated chromosomal anomalies into a direct effect and indirect effect mediated through multiple pregnancies

**Art**	**Total effect**				**Direct effect**				**Indirect effect**				**Estimated size of the indirect effect**
	Unadjusted OR*	**95% CI**	Adjusted§ OR*	**95% CI**	Unadjusted OR*	**95% CI**	Adjusted OR*	**95% CI**	Unadjusted OR*	**95% CI**	Adjusted OR*	**95% CI**	
None	1.0	ref.	1.0	ref.	1.0	ref.	1.0	ref.	1.0	ref.	1.0	ref.	
All methods combined	2.3	1.5 – 3.5	3.2	2.1 – 4.7	1.9	1.2 – 3.0	2.5	1.6 – 3.8	1.2	1.0 – 1.4	1.3	1.1 – 1.5	21.5%
IO^$^ only	1.7	0.9 – 3.1	2.6	1.3 – 5.0	1.5	0.8 – 2.7	2.2	1.2 – 4.0	1.1	1.0 – 1.3	1.2	1.0 – 1.4	18.9%
IVF^£^ only	2.5	1.1 – 6.1	3.4	1.6 – 7.2	1.9	0.8 – 5.0	2.4	1.2 – 4.8	1.3	1.0 – 1.7	1.4	1.0 – 2.0	29.7%
IVF+ICSI^¥^	4.0	1.2 – 13.1	4.4	1.2 – 15.9	3.5	1.1 – 11.5	3.7	1.0 – 13.8	1.1	0.9 – 1.4	1.2	0.9 – 1.5	11.7%
IVF +/- ICSI	2.9	1.6 – 5.4	3.7	2.0 – 7.0	2.3	1.2 – 4.6	2.7	1.5 – 4.9	1.2	1.0 – 1.5	1.4	1.0 – 1.9	23.6%

*Odds ratios (OR) represent the odds of a birth (including live births. stillbirths and pregnancy terminations) with TOF (cases) relative to the odds of a birth with one of the malformed controls (club-foot, angioma, skin abnormality, polydactyly, syndactyly, and congenital hip dislocation).

^§^Adjustment was made for maternal age, occupation, geographic origin, paternal age and year of birth.

^$^Inductors of ovulation.

^£^In vitro fertilization.

^¥^IVF with intracytoplasmic sperm injection.

The estimates were essentially similar to those found for all cases combined (i.e. when cases of TOF with and without associated anomalies were analysed together).

## Discussion

Using population-based data on approximately 400 cases of tetralogy of Fallot (TOF) and 4000 malformed controls, we assessed the previously reported higher risk of TOF [15] that may be due (mediated by) multiple pregnancies in fetuses conceived following assisted reproductive technologies (ART). We used a path-analysis model based on a counterfactual approach in order to decompose the total effect associated with ART into a direct and an indirect effect (i.e. that due to the association between ART and multiple pregnancies).

Our results showed that of the overall 2.6-fold higher odds of TOF in fetuses conceived following ART, 20% was due to the higher likelihood of multiple pregnancies in fetuses conceived following ART (the “indirect effect”). This suggests in turn that multiple pregnancies themselves may be associated with a higher risk of TOF, albeit much less so than ART. The contribution of multiple pregnancies to the higher odds of TOF associated with ART (i.e. the indirect effect) varied according to the method of ART. Our estimates suggested that 30% of the total effect of IVF only was due to multiple pregnancies vs. 11% in the case of IVF + ICSI. The results were similar for analyses restricted to isolated cases of TOF (excluding those associated with chromosomal or other anomalies).

In order to estimate the risk of congenital malformations in multiple pregnancies conceived following ART, previous studies have generally included separate (stratified) analyses for singletons and multiple pregnancies [8,9,12,13,15,32]. This approach can be informative in examining the association between ART and the risk of congenital malformations, in particular regarding the possible existence of an interaction effect between ART and multiple pregnancies. However, these studies did not formally test for statistical significance of any such interaction and had limited power to detect them if they indeed existed. In any case, this approach does not allow a formal estimation of the proportion of the effect of ART on the risk of congenital malformations that may be due to multiple pregnancies.

We found that the “direct” effect associated with ART (i.e. that not mediated by multiple pregnancies) was substantially greater than the indirect effect. This was particularly so in the case of IVF + ICSI. This finding may provide clues as to the underlying mechanisms of the association between ART, particularly for IVF + ICSI, and TOF. The developmental basis of TOF is complex, incompletely elucidated and probably multifactorial [33,34]. The implication of genomic imprinting [35-38] and a role for cardiac neural crest cells in the development of TOF have been suggested [34,39] but any implication of ART in these pathways remains to be shown.

Regarding any effects (the indirect effect) due to twinning, our study was not designed to elucidate what may be the underlying mechanism of any association between twinning and risk of TOF (independently of exposure to ART). In particular, we did not take into account zygosity (or chorionicity), whereas the risk of congenital anomalies in monozygotic twins appears to be higher than that of dizygotic twins [16,17,20]. Our results suggest however, that any effects due to twinning *per se*, whatever its underlying mechanism may be, are likely to be small. Hence, it is possible that twin pregnancies, even in the case of monozygotic twins, are not (or only to a small extent are) associated with a higher risk of TOF, whereas for other CHD twinning *per se* may be a more important risk factor [16,17,20].

We also did not account for any potential effect of the “vanishing” twin syndrome on our estimates. Several studies have found that ART multiple pregnancies in which a twin vanished had poorer obstetrical and perinatal outcomes [40-43]. However, to our knowledge, there is no documented association between the vanishing twin syndrome and risk of birth defects in general or of CHD in particular. If indeed such an association exists in the case of TOF, misclassification of vanishing twin pregnancies as singletons would result in an underestimation of the mediating effect of multiple pregnancies in the association between ART and risk of TOF.

Our study has certain limitations. Even if the total number of cases of TOF and controls was fairly large, the numbers of cases exposed to the different methods of ART were small and hence confidence interval were fairly wide reflecting the relative imprecision of our estimates of ORs particularly for specific techniques of ART.

Although we chose frequent and heterogeneous malformations for which no association with ART exist in the literature, bias due to the association between ART and malformations in the control group cannot be excluded. In particular, if ART were associated with a higher risk of one or more of the malformations in the control group, the consequence would be an underestimation of the true association between ART and TOF.

Due to a lack of available information in our data, we did not adjust for certain maternal conditions including pregestational or gestational diabetes and obesity, tobacco, alcohol and drug consumption. Although these factors may be associated with risk of birth defects in general, or of CHD in particular, their associations, if any, with the risk of specific CHD, including TOF are generally not known.

We also could not adjust for maternal folic acid/multivitamins use that is associated with a lower risk of CHD [44] and in addition is more frequent in women who conceive following ART [13,45]. Hence, we cannot rule out residual confounding due to risk (or protective) factors associated with ART and/or twinning that were not taken into account.

In addition, our study was not designed to and cannot disentangle the effects that may be due to infertility of couples vs. ART or twinning *per se*[4,9,46]. Some authors have found that the risk of birth defects is increased in infertile women who conceive spontaneously, which suggests that the underlying infertility problems may explain part or perhaps all of the association between ART and risk of birth defects. However, the strategy of “adjusting for infertility” (time to pregnancy or duration of infertility) has been criticized [5] as it could be “synonymous” with exposure to ART. In any case, in our data, essentially all women who had a duration of infertility of more than two years had conceived following ART; adjustment for infertility duration was therefore not feasible in our study.

The frequency of missing data was low for exposure to ART and maternal characteristics. However, paternal age was missing for 25% of the study population. We adjusted for paternal age as it can be related to ART exposure and particularly ICSI. Results were nevertheless similar when models did not include paternal age and in analyses that included multiple imputation for paternal age (results not shown, available from authors).

## Conclusion

In conclusion, we found that most of the higher risk of TOF associated with ART is a “direct” effect (i.e. not mediated by multiple pregnancies) and only a small proportion of the effect may be due to multiple pregnancies. This was particularly the case for IVF + ICSI and may provide clues about the possible underlying mechanism(s) for the association between ART and TOF. The path-analysis approach presented here may be more generally useful for assessing the role of multiple pregnancies in the association between ART and risk of other congenital malformations or other adverse outcomes.

## Competing interests

The authors declare that they have no competing interest.

## Authors’ contributions

BK conceived the study. KT conducted the main statistical analyses and wrote the first draft of the manuscript with BK. NL assisted with statistical analysis. LH, DB and FG contributed to the conceptualization of ideas and made suggestions about the required analyses. LH and DB provided expertise as pediatric cardiologists. All of the authors contributed to the interpretation of findings and revisions of the article. All authors read and approved the final manuscript.

## EPICARD study group

*Principal Investigators:* François Goffinet, Babak Khoshnood

*Steering Committee:* Damien Bonnet (Hôpital Necker Enfants Malades, AP-HP, Centre de référence M3C, Université Paris Descartes, Paris)

Johanna Calderon (INSERM U953)

Drina Candilis (Université Paris-Diderot, Paris)

Anne-Lise Delezoide (Hôpital Robert Debré, AP-HP, Service de biologie du Développement, Université Paris-Diderot, Paris)

Catherine De Vigan (INSERM U953, Paris)

François Goffinet (Groupe Hospitalier Cochin-Hôtel Dieu, AP-HP, Maternité Port-Royal et INSERM U953, Université Paris Descartes, Paris)

Lucile Houyel (Hôpital Marie Lannelongue, Service de chirurgie des cardiopathies congénitales, Le Plessis-Robinson)

Jean-Marie Jouannic (Hôpital Trousseau, AP-HP, Centre pluridisciplinaire de diagnosticprénatal, UPMC, Paris)

Babak Khoshnood (INSERM U953, Paris)

Nathalie Lelong (INSERM U953, Paris)

Suzel Magnier (Hôpital Robert Debré, AP-HP, Service de cardiologie, Paris)

Jean-François Magny (Institut de Puériculture et de périnatologie, Service de néonatologie, Paris)

Caroline Rambaud (Hôpital Raymond Poincarré, AP-HP, Service d’anatomie et cytologie pathologiques – Médecine légale, UVSQ, Garches)

Dominique Salomon (INSERM U953, Paris)

Véronique Vodovar (INSERM U953, Paris)

*Project Coordination and Data Analysis Committee:* François Goffinet, Babak Khoshnood, Nathalie Lelong, Anne-Claire Thieulin, Thibaut Andrieu, Véronique Vodovar

*Independent Data Monitoring Committee (URC Paris Centre et CIC Cochin Necker Mère Enfant):* Maggy Chausson, Anissa Brinis, Laure Faure, Maryline Delattre, Jean-Marc Treluyer (Groupe Hospitalier Cochin-Hôtel Dieu, AP-HP, Université Paris Descartes, Paris)

*External Scientific Committee:* Gérard Bréart, Dominique Cabrol, Alain Sérraf, Daniel Sidi, Marcel Voyer

*Participating Centers:* The Greater Paris Area (Paris and its surrounding suburbs) public (AP-HP) and private maternity units, Departments of Pediatric Cardiology and Pediatric Cardiac Surgery, pediatric cardiologists in private practice, Neonatal Intensive Care Units, Pediatric Intensive Care Units, Emergency Transfer Services (SMUR), Departments of Pathology, Sudden Death Centers, Departments of Family and Infant Protection (DFPE)
